# Zero-Sum Matrix Game with Payoffs of Dempster-Shafer Belief Structures and Its Applications on Sensors

**DOI:** 10.3390/s17040922

**Published:** 2017-04-21

**Authors:** Xinyang Deng, Wen Jiang, Jiandong Zhang

**Affiliations:** School of Electronics and Information, Northwestern Polytechnical University, Xi’an 710072, China; jdzhang@nwpu.edu.cn

**Keywords:** sensor selection, intrusion detection, matrix game, imprecise payoff, Dempster–Shafer evidence theory, belief function

## Abstract

The zero-sum matrix game is one of the most classic game models, and it is widely used in many scientific and engineering fields. In the real world, due to the complexity of the decision-making environment, sometimes the payoffs received by players may be inexact or uncertain, which requires that the model of matrix games has the ability to represent and deal with imprecise payoffs. To meet such a requirement, this paper develops a zero-sum matrix game model with Dempster–Shafer belief structure payoffs, which effectively represents the ambiguity involved in payoffs of a game. Then, a decomposition method is proposed to calculate the value of such a game, which is also expressed with belief structures. Moreover, for the possible computation-intensive issue in the proposed decomposition method, as an alternative solution, a Monte Carlo simulation approach is presented, as well. Finally, the proposed zero-sum matrix games with payoffs of Dempster–Shafer belief structures is illustratively applied to the sensor selection and intrusion detection of sensor networks, which shows its effectiveness and application process.

## 1. Introduction

Uncertainty extensively exists in numerous scientific and engineering fields. Among a variety of fundamental problems related to uncertainty, how to express the uncertain information is one of the first issues. Usually, uncertainty contains randomness, fuzziness, ambiguity, and so on. From the perspective of semantics or linguistics, Klir and Yuan [1] have identified three basic types: fuzziness, discord and non-specificity, where fuzziness stands for the unclearness or indistinctness about objects’ characters, discord in the conflict or randomness to an object and non-specificity in the diversity of possible results. The discord and non-specificity are unified as ambiguity [2]. Probability theory, fuzzy set [3], rough set [4], Dempster–Shafer evidence theory [5,6] and the maximum-entropy approach [7,8], to name but a few [9], are some of representative theories or approaches to deal with uncertain information. Especially, due to providing feasible ways to represent and synthesize uncertain information, Dempster–Shafer evidence theory has attracted increasing interest in many fields, such as surveillance [10], intrusion detection [11], etc. As attempts to improve conventional Dempster–Shafer evidence theory, generalized evidence theory [12] and D numbers [13,14,15,16,17,18] have also received researchers’ attention recently. Among them, the model of D numbers, which generalizes belief structures, is a relatively novel means to represent uncertain information by overcoming the exclusiveness hypothesis and completeness constraint in Dempster-Shafer theory.

Apart from uncertainty, which depicts the essential characteristic of information, things are universally interrelated and interact with each other. The interactive relationship among things or objects, either competitive or cooperative, arises in countless types of fields. Game theory gives a mathematical framework to study the interactions among related individuals in interactive decision situations where the aims, goals and preferences of the participating agents are potentially in conflict [19,20,21] and has achieved great success in multiple of disciplines and fields [22,23,24,25,26]. As a game model representing the most conflicting and competitive relationship, the two-person zero-sum game models the case where players’ interests are completely opposed [27]. As a special case of that game, the two-person zero-sum matrix game, short for the matrix game representing a pervasive interactive relationship, has been addressed since von Neumann and Morgenstern [28] published their seminal book *Theory of Games and Economic Behavior*.

Usually, in a matrix game, payoffs are in the form of crisp values representing utilities received by players. However, as mentioned above, uncertainty widely exists, which may result in the difficulty to obtain deterministic payoffs. Instead, uncertain or imprecise payoffs in matrix games are more universal and practical in reality, since it is unavoidable in most cases to involve subjective or objective uncertainty caused by many reasons in constructing the payoff matrix of a matrix game. Therefore, investigation of matrix games with imprecise or uncertain payoffs is very important and of great significance [29]. In previous studies, zero-sum matrix games with payoffs of interval-valued [30,31,32,33,34], or fuzzy data [35,36,37,38], or rough sets [39], or intuitionistic fuzzy sets [40,41,42], or interval-valued intuitionistic fuzzy sets [43] have been given much attention. In addition, other forms of payoff matrices, such as ones with fuzzy random variables [44] or lattice values [45], have also received some attention.

Compared with the rich studies on interval- or fuzzy-based matrix games, imprecise matrix games with payoffs involving the uncertainty of ambiguity (i.e., discord and non-specificity) have been given less attention. Existing representative studies are included in [46,47,48], where Dempster–Shafer evidence theory is employed to express ambiguity. In most cases, payoffs are on the basis of linguistic variables or assessments. For example, in [46,47], the initial payoffs are all in the form of categorical variable-based belief structures, where the involved categorical variables represent the ordered levels of reflecting the consequences of players’ actions. When dealing with the game consisting such payoffs, the payoffs are transformed into expected utility intervals based on each categorical variable’s utility function; in the process, the original ambiguity has been changed to the interval uncertainty. What is more, in [48], the payoffs have been directly transformed to crisp numbers that do not contain uncertainty. In contrast, this paper immediately studies the games with ambiguity based on conventional numerical payoffs, but does not need to utilize additionally utility functions transforming categorical assessment levels to real numbers. In order to implement the goal, the Dempster–Shafer belief structure defined on the real line is used to represent this kind of uncertain payoff. As a result, a zero-sum matrix game with payoffs of Dempster–Shafer belief structures is constructed. Within the paper, a decomposition method is proposed to solve the game. The obtained value of this game is still in the form of belief structures, which maintains the consistency between the payoff matrix and the value of the game. Besides, an alternative solution to solve the game is presented in the paper to overcome the possible issue of high computational complexity caused by the decomposition method. Finally, this novel game model is illustratively applied to the sensor selection and intrusion detection of sensor networks to show its potential applications and effectiveness.

The rest of the paper is organized as follows. Section 2 gives a brief introduction about Dempster–Shafer evidence theory and belief structures defined on the real line. Section 3 gives the formal representation of a two-person zero-sum matrix game and its extension of Dempster–Shafer belief structures. Then, the method to solve the game is proposed in Section 4, and a Monte Carlo-based alternative solution is presented in Section 5. Section 6 gives the applications of the proposed model and method in the sensor selection and intrusion detection of sensor networks. At last, Section 7 concludes this paper.

## 2. Preliminaries

### 2.1. Basics of Dempster-Shafer Evidence Theory

Dempster–Shafer evidence theory [5,6], also called Dempster–Shafer (D-S) theory or evidence theory, is a popular theory to deal with uncertain information [49,50,51,52,53,54,55,56]. Having a similar effect of aggregating operators [57,58,59,60] and as the counterpart of the probability-based approaches [61,62,63], such as composite hypothesis testing, Bayesian approaches and the maximum entropy approach, D-S theory is another widely-used framework for multi-sensor information fusion [64,65,66,67,68,69,70]. Compared with traditional probability theory, this theory has the advantage of directly expressing the “uncertainty” by assigning the probability to the set composed of multiple objects; it therefore has attracted increasing interest in uncertainty reasoning and modeling.

In D-S theory, the uncertainty is modeled by belief structures. A belief structure is formally expressed by a mapping $m:2^{\Omega }\to \left[0,1\right]$, such that:

(1) $$\begin{array}{c} m\left(\emptyset \right)=0\;and\;\sum_{A\in 2^{\Omega }}m(A)=1, \end{array}$$

where Ω is called the frame of discernment, which is a finite nonempty set, $2^{\Omega }$ denotes the power set of Ω and set *A* with m(A)>0 is called a focal element. A belief structure is also called a mass function or a basic probability assignment (BPA). The belief measure and plausibility measure, denoted as Bel and Pl, respectively, are two important measures associated with the belief structure; they are defined by:

(2) $$Bel\left(A\right)=\sum_{B\subseteq A}m(B),$$

and:

(3) $$Pl\left(A\right)=1-Bel\left(\bar{A}\right)=\sum_{B\cap A\neq \emptyset }m(B),$$

where $\bar{A}=\Omega -A$. For every A∈Ω, Bel(A)≤Prob(A)≤Pl(A) is satisfied. The measures Bel and Pl quantify the lower limit and upper limit of the support to focal element *A*, respectively.

### 2.2. D-S Belief Structures Defined on the Real Line

In most cases, a D-S belief structure is defined over a discrete nonempty set. Some pioneering researchers have investigated the case where the focal elements of belief structures are closed intervals, which leads to an extension of D-S theory to the real line [−∞,+∞]. Please refer to [71,72,73] for more details. A D-S belief structure defined on the real line has the form:

(4) $$\sum_{i}m(F_{i})=1\;,\;F_{i}=\left[a_{i},b_{i}\right]\subseteq \left[-\infty ,+\infty \right]$$

where $F_{i}$ is the focal element of belief structure *m*.

Given a D-S belief structure defined on the real line, Yager [71] has presented an approach to construct its cumulative distribution function (CDF). The CDF has graphically shown the lower and upper distributions of the associated belief structure. Let $L_{x}$ be a subset of R whose elements are less than or equal to *x*, namely $L_{x}=\left\{z|z\leq x\right\}$, the CDF of a belief structure *m*, denoted as ${CDF}_{m}\left(x\right)$, is ${CDF}_{m}\left(x\right)=\left[Bel_{m}\left(L_{x}\right),\;Pl_{m}\left(L_{x}\right)\right]$, satisfying:

(5) $$Bel_{m}\left(L_{x}\right)=\sum_{i,\;F_{i}\subseteq L_{x}}m(F_{i})=\sum_{i,\;b_{i}\leq x}m(F_{i}),$$

(6) $$Pl_{m}\left(L_{x}\right)=\sum_{i,\;F_{i}\cap L_{x}\neq \emptyset }m(F_{i})=\sum_{i,\;a_{i}\leq x}m(F_{i}).$$

**Figure 1:**
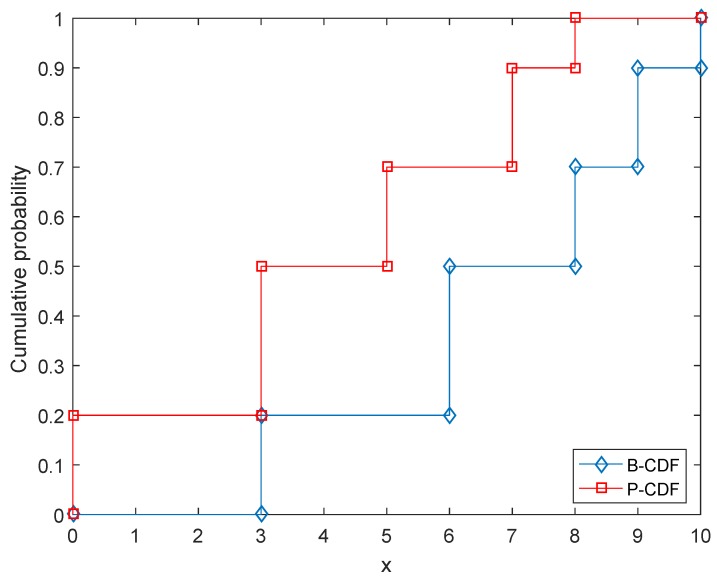
Example of a D-S belief structure’s CDF with respect to its associated variable *x*.

For simplicity, the low distribution calculated by $Bel_{m}\left(L_{x}\right)$ is called B-CDF; the upper distribution calculated by $Pl_{m}\left(L_{x}\right)$ is called P-CDF. An example of a belief structure’s CDF is given in Figure 1, in which the associated belief structure is m([0,3])=0.2, m([3,6])=0.3, m([5,8])=0.2, m([7,9])=0.2, m([8,10])=0.1.

## 3. Two-Person Zero-Sum Matrix Game and Its Extension of D-S Belief Structures

In a two-person zero-sum matrix game, there are two players; each has a finite number of strategies to select in each play of the game. Assume that $S_{1}=\left\{\delta_{1},\delta_{2},\cdots ,\delta_{p}\right\}$ and $S_{2}=\left\{\sigma_{1},\sigma_{2},\cdots ,\sigma_{q}\right\}$ are the sets of pure strategies for Players 1 and 2. The vectors $x=(x_{1},x_{2},\cdots ,x_{p})$ and $y=(y_{1},y_{2},\cdots ,y_{q})$ are known as the mixed strategies of Players 1 and 2, respectively, where $\sum_{i=1}^{p}x_{i}=1,x_{i}\geq 0$, and $\sum_{j=1}^{q}y_{j}=1,y_{j}\geq 0$.

The classical zero-sum matrix game is based on a deterministic payoff matrix. Let the payoff matrix of Player 1 be denoted as $A={\left(a_{ij}\right)}_{p\times q}$, which is a real matrix, then a two-person zero-sum matrix game is expressed by ({x},{y},A) or *A* simply. In matrix game *A*, if Player 1 selects strategy x and Player 2 chooses strategy y, the expected gain (also called utility) of Player 1 can be computed as:

(7) $$u_{1}\left(x,y\right)=xAy^{T}=\sum_{i=1}^{p}\sum_{j=1}^{q}x_{i}a_{ij}y_{j}.$$

Correspondingly, in this condition, the expected loss of Player 2 is $u_{2}\left(x,y\right)=-xAy^{T}$. The game reaches a Nash equilibrium if the strategy combination $\left(x^{*},y^{*}\right)$ selected by players satisfies:

(8) $$\left\{\begin{array}{c} u_{1}\left(x^{*},y^{*}\right)\geq u_{1}\left(x,y^{*}\right),\;\forall x\\ u_{2}\left(x^{*},y^{*}\right)\geq u_{2}\left(x^{*},y\right),\;\forall y \end{array}\right.$$

In order to find the optimal strategies $x^{*}$ and $y^{*}$, von Neumann presented the minimax theorem to solve the game. According to that theorem, a player should find the mixed strategy that maximizes the minimum expected gain, or equivalently, a player should select the mixed strategy that minimizes the maximum expected loss. In the mathematical formulation, the criterion is expressed by a pair of primal-dual linear programming problems as below:

(9) $$\begin{array}{c} \max\;v\\ s.t.\left\{\begin{array}{c} \sum_{i=1}^{p}a_{ij}x_{i}^{*}\geq v,\;j=1,\cdots ,q\\ \sum_{i=1}^{p}x_{i}^{*}=1\\ x_{i}^{*}\geq 0,\;i=1,\cdots p \end{array}\right. \end{array}$$

and:

(10) $$\begin{array}{c} \min\;w\\ s.t.\left\{\begin{array}{c} \sum_{j=1}^{q}a_{ij}y_{j}^{*}\leq w,\;i=1,\cdots ,p\\ \sum_{j=1}^{q}y_{j}^{*}=1\\ y_{j}^{*}\geq 0,\;j=1,\cdots q \end{array}\right. \end{array}$$

In that case, $x^{*}$ and $y^{*}$ are the optimal strategies, and V(=v=w) is called the value of the matrix game *A*.

As shown above, the traditional zero-sum matrix game is on the basis of deterministic payoffs. However, in the real world, the game may be composed by uncertain payoffs because of the varieties of uncertain factors. In previous studies, matrix games with payoffs of interval numbers, fuzzy numbers, intuitionistic fuzzy sets, and so on, have received much concern. The matrix game with belief structure payoffs, which represent the ambiguity involved in players’ utilities, has been paid less attention. In the paper, the deterministic zero-sum matrix game is extended to the uncertain environment expressed by D-S belief structures over the real line.

Modeled after the zero-sum matrix game within the environment of deterministic payoffs, a zero-sum matrix game with D-S belief structure payoffs is represented as a triplet ({x},{y},M) or *M* simply, where x and y are the strategies of two players, and *M* is the payoff matrix consisting of belief structures defined over the real line. Formally, *M* is expressed as:

(11) $$M=\left[\begin{array}{cccc} m_{11} & m_{12} & \cdots & m_{1q}\\ m_{21} & m_{22} & \cdots & m_{2q}\\ \vdots & \vdots & \ddots & \vdots \\ m_{p1} & m_{p2} & \cdots & m_{pq} \end{array}\right].$$

## 4. Proposed Method to Solve Zero-Sum Matrix Games with D-S Belief Structure Payoffs

Once the model of the zero-sum matrix game with D-S belief structure payoffs has been proposed, how to solve the game becomes the following problem. Herein, a basic fact or hypothesis must be clarified. In the research of uncertain matrix games, the term “solve the game” does not mean obtain a unique combination of optimal strategies, i.e., a Nash equilibrium. In fact, in the uncertain environment, the deterministic Nash equilibrium basically cannot be derived due to the impact of uncertain payoffs. This will yield different Nash equilibria associated with different cases of payoffs, and any single equilibrium is trivial. Instead, solving the game is mainly to calculate the value of the game, which is uncertain in an uncertain matrix game.

As for the zero-sum matrix game with D-S belief structure payoffs, a decomposition method is proposed to calculate the value of the game via decomposing it into multiple zero-sum matrix games with interval data. The details of the method are shown as follows. It mainly contains the steps “decompose”-“calculate”-“compose”.

**Figure 2:**
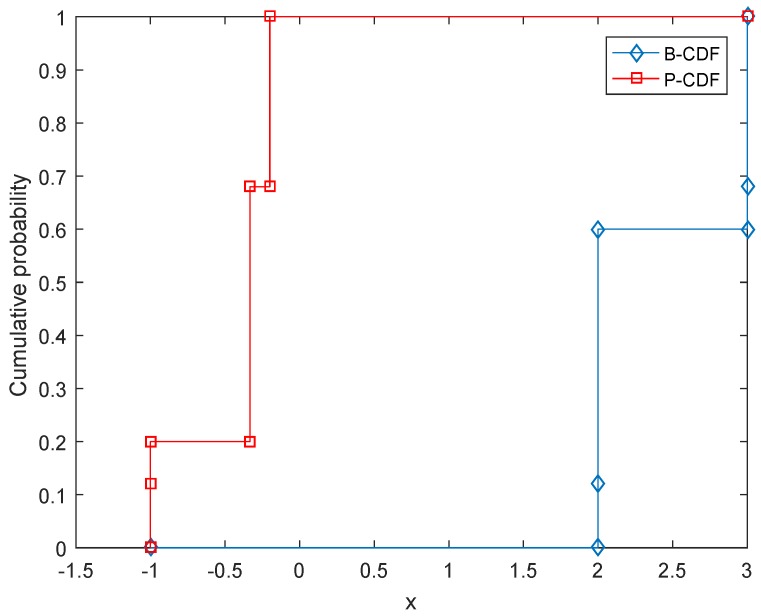
The CDF of the value of the game given in Table 1 with respect to the associated variable *x*.

- “Decompose”: A zero-sum matrix game with D-S belief structure payoffs ({x},{y},M), where $M={\left[m_{ij}\right]}_{p\times q}$ with i∈{1,⋯,p} and j∈{1,⋯,q}, is decomposed into $\prod_{i,j}\mathrm{NumFocals}(m_{ij})$ zero-sum matrix games with interval data $\left(\left\{x\right\},\left\{y\right\},I={\left[I_{kl}\right]}_{p\times q}\right)$, k∈{1,⋯,p} and l∈{1,⋯,q}, where $\mathrm{NumFocals}(m_{ij})$ is the number of focal elements in belief structure $m_{ij}$, and $I_{kl}$ is a focal element extracting from $m_{ij}$. Each $I={\left[I_{kl}\right]}_{p\times q}$ has a belief degree indicated by BelD(I) to express its probability of occurrence, which is determined by all $I_{kl}$ in *I*. For example, Table 1 shows a zero-sum matrix game with D-S belief structure payoffs, which can be decomposed into the following four zero-sum matrix games with interval data:
  $I_{1}=\left[\begin{array}{cc} \left[1,2\right] & \left[-1,3\right]\\ \left[-1,0\right] & \left[-1,1\right] \end{array}\right]$ with belief degree $BelD(I_{1})=0.6\times 1\times 1\times 0.2=0.12$.
  $I_{2}=\left[\begin{array}{cc} \left[1,2\right] & \left[-1,3\right]\\ \left[-1,0\right] & \left[0,2\right] \end{array}\right]$ with belief degree $BelD(I_{2})=0.6\times 1\times 1\times 0.8=0.48$.
  $I_{3}=\left[\begin{array}{cc} \left[3,5\right] & \left[-1,3\right]\\ \left[-1,0\right] & \left[-1,1\right] \end{array}\right]$ with belief degree $BelD(I_{3})=0.4\times 1\times 1\times 0.2=0.08$.
  $I_{4}=\left[\begin{array}{cc} \left[3,5\right] & \left[-1,3\right]\\ \left[-1,0\right] & \left[0,2\right] \end{array}\right]$ with belief degree $BelD(I_{4})=0.4\times 1\times 1\times 0.8=0.32$.
- “Calculate”: In this step, the values of obtained interval-valued matrix games are calculated. At present, there are many existing approaches to solve a zero-sum matrix game with interval data. A well-developed approach proposed by Liu and Kao [34] is employed to solve these interval-valued matrix games in the paper. Given a zero-sum matrix game with interval data $\left(\left\{x\right\},\left\{y\right\},I={\left[I_{kl}\right]}_{p\times q}\right)$, where $I_{kl}=\left[I_{kl}^{L},I_{kl}^{R}\right]$, k∈{1,⋯,p} and l∈{1,⋯,q}, according to Liu and Kao’s approach, the lower bound of the value of the game, denoted as $\underline{V}$, is calculated by:
  (12) $$\begin{array}{c} \max\;\underline{v}\\ s.t.\left\{\begin{array}{c} \sum_{k=1}^{p}I_{kl}^{L}x_{k}\geq \underline{v},\;l=1,\cdots ,q\\ \sum_{k=1}^{p}x_{k}=1\\ x_{k}\geq 0,\;k=1,\cdots p \end{array}\right. \end{array}$$
  and:
  (13) $$\begin{array}{c} \min\;\underline{w}\\ s.t.\left\{\begin{array}{c} \sum_{l=1}^{q}I_{kl}^{L}y_{l}\leq \underline{w}\;,\;k=1,\cdots ,p\\ \sum_{l=1}^{q}y_{l}=1\\ y_{l}\geq 0,\;l=1,\cdots q \end{array}\right. \end{array}$$
  $\underline{V}=\underline{v}=\underline{w}$. The upper bound of the value of the game, denoted as $\bar{V}$, is calculated by:
  (14) $$\begin{array}{c} \max\;\bar{v}\\ s.t.\left\{\begin{array}{c} \sum_{k=1}^{p}I_{kl}^{R}x_{k}\geq \bar{v},\;l=1,\cdots ,q\\ \sum_{k=1}^{p}x_{k}=1\\ x_{k}\geq 0,\;k=1,\cdots p \end{array}\right. \end{array}$$
  and:
  (15) $$\begin{array}{c} \min\;\bar{w}\\ s.t.\left\{\begin{array}{c} \sum_{l=1}^{q}I_{kl}^{R}y_{l}\leq \bar{w}\;,\;k=1,\cdots ,p\\ \sum_{l=1}^{q}y_{l}=1\\ y_{l}\geq 0,\;l=1,\cdots q \end{array}\right. \end{array}$$
  $\bar{V}=\bar{v}=\bar{w}$. For example, the values of the four interval-valued matrix games associated with Table 1 are calculated. For $I_{1}$, the value is [−1,2] with belief degree 0.12. The value of $I_{2}$ is [−1/3,2] with belief degree 0.48. The value of $I_{3}$ is [−1,3] with belief degree 0.08. The value of $I_{4}$ is [−0.2,3] with belief degree 0.32.
- “Compose”: In this step, the values calculated by the above step are composed to form an overall value for the zero-sum matrix game with belief structure payoffs. Let us still use the game shown in Table 1 as the example. According to the step “calculate”, the values of interval-valued matrix games associated with the game shown in Table 1 are [−1,2] with belief degree 0.12, [−1/3,2] with belief degree 0.48, [−1,3] with belief degree 0.08 and [−0.2,3] with belief degree 0.32, respectively. Therefore, the value of the matrix game shown in Table 1 is m([−1,2])=0.12, m([−1/3,2])=0.48, m([−1,3])=0.08, m([−0.2,3])=0.32. This value is also in the form of D-S belief structures; its CDF is shown in Figure 2.

Via the steps shown above, the value of a zero-sum matrix game with belief structure payoffs is calculated. This value indicates the distribution of equilibrium payoffs of the game.

## 5. Alternative Solution: A Monte Carlo Simulation Approach Based on the Latin Hypercube Sampling

Essentially, the proposed decomposition method presented in Section 4 enumerates all possible interval-valued matrix games of the associated zero-sum matrix game with belief structure payoffs. It will generate $\prod_{i,j}\mathrm{NumFocals}(m_{ij})$ zero-sum interval-valued matrix games. This would not be an issue in many cases. However, if the size of a game’s payoff matrix and the number of focal elements in belief structures are simultaneously large, the amount of generated interval-valued matrix games will be very large. For example, Table 2 shows a 5×3 zero-sum matrix game with D-S belief structure payoffs. In the game, each payoff is a belief structure having four focal elements. If using the decomposition method presented in Section 4, it has to generate and deal with $4^{15}=$ 1,073,741,824 zero-sum interval-valued matrix games. If we run the corresponding MATLAB code on the MATLAB R2016a software, which has been installed on a personal computer with Intel Pentium CPU G3250 (3.20 GHz, two cores) and 8 GB RAM, the time for solving a zero-sum interval-valued matrix game is 0.0141 s, which is the average of running 10,000 times. As a result, solving $4^{15}$ zero-sum interval-valued matrix games will take $0.0141\times 4^{15}\approx$ 15,140,000 s, which is more than 175 days. Therefore, it is quite computation-intensive.

**Figure 3:**
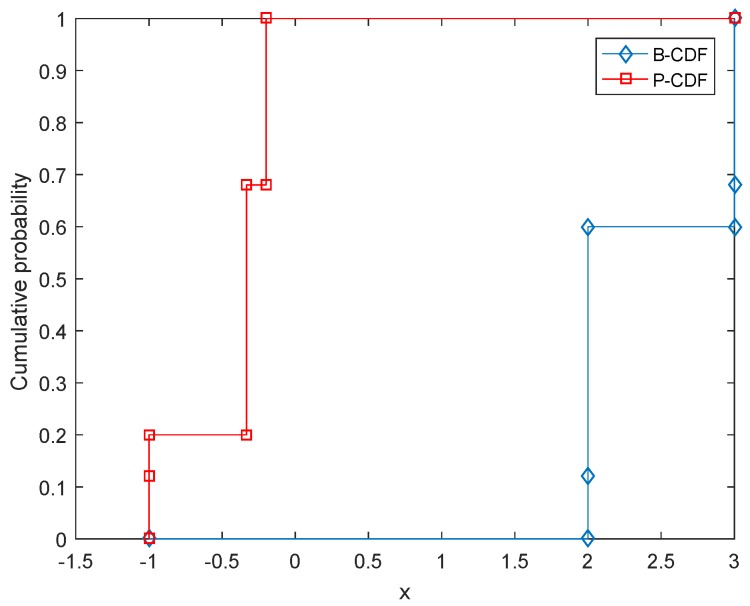
Obtained CDF of the value of the game shown in Table 1 with respect to the associated variable *x* by using the LHS-based Monte Carlo simulation while sampling size *T* = 10,000.

**Figure 4:**
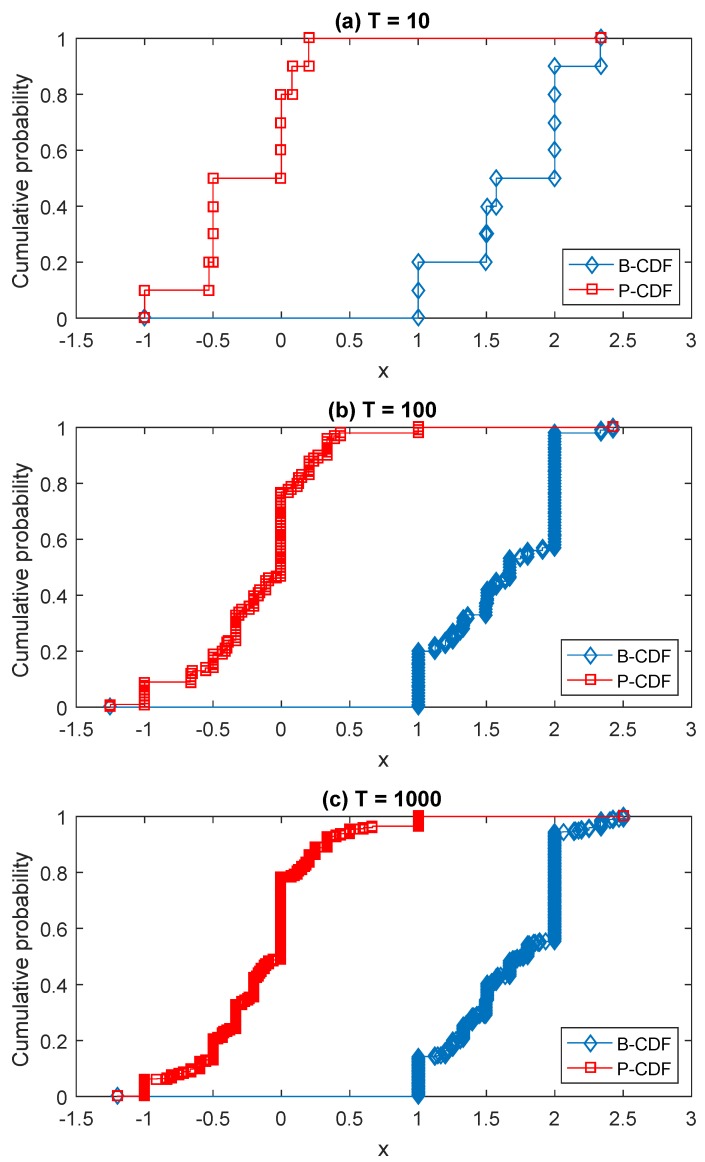
CDF of the value of the game shown in Table 2 with respect to the associated variable *x* by using the LHS-based Monte Carlo simulation with different sampling sizes *T*.

Facing this situation, as an alternative solution, a Monte Carlo simulation based on the Latin hypercube sampling (LHS) can be used, whose process is shown in Algorithm 1. LHS [74] is a popular sampling method to generate a near-random sample of parameter values from a multidimensional distribution [75,76]. By using the LHS, an interval-valued matrix game is generated according to the original matrix game with belief structure payoffs in every sampling, then the value of the interval-valued matrix game, which is an interval, can be calculated. Given a predetermined sampling size *T*, the obtained *T* intervals can be used to construct the distribution of the value of the game. This distribution can also be seen as a belief structure, which could be graphically shown in a CDF figure in terms of Equations (5) and (6). For example, Figure 3 shows the CDF of the value of the game given in Table 1 obtained by using the LHS-based Monte Carlo simulation approach. It is basically the same as Figure 2. The comparison verifies the result obtained by using the proposed decomposition method in Section 4 and simultaneously shows the effectiveness of these two methods. Besides, now the game shown in Table 2 can be handled by using the LHS-based Monte Carlo simulation approach; the results are shown in Figure 4. By using the alternative solution, for the game shown in Table 2, we do not need to solve 1,073,741,824 zero-sum interval-valued matrix games, but just deal with *T* interval-valued matrix games, which could greatly reduce the amount of computation. As shown in Figure 4, the obtained B-CDF and P-CDF become more and more precise with the rise of sampling size *T*. In the concrete applications, the value of *T* can be determined according to the requirement of precision and computing capability. Note that since the Monte Carlo simulation is an approximate approach that cannot achieve 100% precision, it is suggested to be used when the amount of computation is very large and the computing capability is limited.

By comparing the proposed decomposition method presented in Section 4 and the LHS-based Monte Carlo simulation approach presented in this section, we can find that: (i) in the facet of the optimality or precision property, the decomposition method is an accurate method that would obtain the exact value of the game with belief structure payoffs, in contrast to the Monte Carlo simulation approach being a sampling-based approach, which only derives the approximate result; (ii) in the aspect of complexity, the decomposition method has a bearable computational complexity if the game and its payoffs are relatively simple; when the game becomes complicated, the amount of computation of using the decomposition method will rapidly increase, at that time the Monte Carlo simulation approach is suggested to reduce the computation; (iii) in the aspect of parallelization, both proposed methods can be performed in parallel, which provides a potential way to accelerate the computing process when the amount of computation is extremely large.
**Algorithm 1:** The Latin hypercube sampling (LHS)-based Monte Carlo simulation approach to solve a zero-sum matrix game with D-S belief structure payoffs. **INPUT**: Zero-sum matrix game with D-S belief structure payoffs ({x},{y},M),   where $M={\left[m_{ij}\right]}_{p\times q}$ with i∈{1,⋯,p} and j∈{1,⋯,q}; Sampling size *T* **OUTPUT**: B-CDF, P-CDF Generate a *T*-by-(p×q) matrix *L* containing a LHS of *T* values on each of (p×q) variables; **FOR**
*k* = 1 : stepsize 1 : *T*   **FOREACH**
$m_{ij}$ in *M*     Get a focal element $F_{\left(ij\right)}\in \mathrm{FocalElements}\left(m_{ij}\right)$ in terms of L(k,i,j);   **END**   Generate an interval-valued matrix game $I_{k}={\left[F_{\left(ij\right)}\right]}_{p\times q}$ according to all $F_{\left(ij\right)}$;   Solve $I_{k}={\left[F_{\left(ij\right)}\right]}_{p\times q}$ based on Equations (12)–(15), the obtained value is denoted as $\left[{\underline{V}}_{k},{\bar{V}}_{k}\right]$; **END** Once having all $\left[{\underline{V}}_{k},{\bar{V}}_{k}\right]$, k=1,⋯,T, then  (i) calculate the B-CDF according to Equation (5);  (ii) calculate the P-CDF according to Equation (6); where $m(\left[{\underline{V}}_{k},{\bar{V}}_{k}\right])=1/T$.

## 6. Applications

### 6.1. An Illustrative Application in Sensor Selection

In this subsection, an application of sensor selection adapted from [27] is used to show the process of applying the proposed zero-sum matrix game with belief structure payoffs and its solution method.

**Figure 5:**
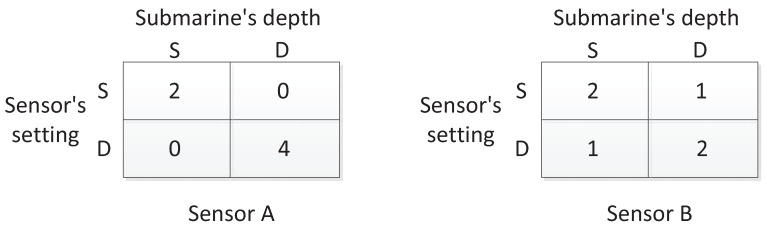
The detection ranges of Sensors A and B in the form of crisp numbers.

In [27], the author presented an example of applying zero-sum matrix games with crisp payoffs to sensor selection. Suppose there are two types of sensors A and B, and one of the two sensors is acquired for use in detecting submarines. Note that sensors with different buoyancy submerge to different depths; hence, each type of sensors works at a certain depth, and a sensor network consisting of sensors belonging to the same type is approximatively deployed on a two-dimensional plane. Both of the sensors have “shallow” (S) and “deep” (D) settings or modes, but Sensor B’s long-range detection capability is weakened in order to enhance its robustness; the capacity that the detection range is not zero even if the submarine’s depth (which is also either S or D) is guessed incorrectly. The detection ranges of the two sensors are shown in Figure 5. The question is which kind of sensor is more effective.

**Figure 6:**
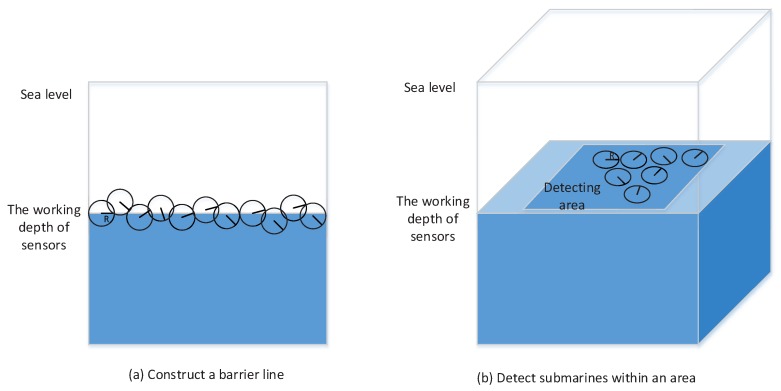
Two cases in sensor selection for submarine detection.

**Figure 7:**
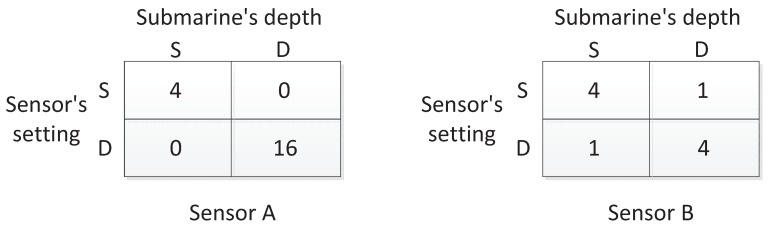
Squared detection ranges for Sensors A and B in the form of crisp numbers.

In order to answer the question, in [27], the author has analyzed two cases, as shown in Figure 6. One is that the sensors are used to construct a linear barrier. In that case, each matrix in Figure 5 is regarded as the payoff matrix in a game, and the value of the game is an equivalent detection range. Let $V_{A}$ and $V_{B}$ be the values of Sensor A and Sensor B games. According to the minimax criterion shown in Equations (9) and (10), it is calculated that $V_{A}=1.333$ and $V_{B}=1.5$. Therefore, Sensor B is better in this case. The other case is to perform the task that detects hidden submarines within a large area of size *C*. In the case, if *n* sensors are used, the fraction of submarines detected can be calculated by ∑i=1nπRi2∑i=1nπRi2CC where $R_{i}$ is the detection range of the *i*-th sensor. On average, the ratio is positively related to $E(R^{2})$, which is the value of the game with squared detection ranges. For Sensors A and B, the games become new forms as shown in Figure 7, which are the same as those in Figure 5, but each entry has been squared since now the task is to detect submarines within an area. In the case, the values of new sensor games becomes $V_{A}^{\prime }=3.2$ and $V_{B}^{\prime }=2.5$. Therefore, Sensor A is better for the task of submarine detection within a large area.

**Figure 8:**
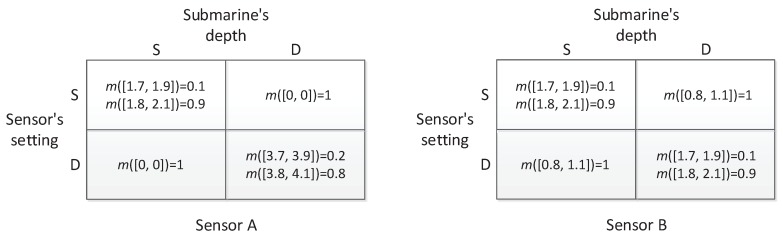
The detection ranges of Sensors A and B in the form of D-S belief structures.

The analysis given above is on the basis of deterministic payoff matrices of sensor games. However, in reality, due to the complexity of the marine environment, the sensor’s reliability and the opponent’s interference, the detection ranges of sensors are not precise, but have a degree of uncertainty. In the paper, the uncertainty is modeled by D-S belief structures. Suppose the detection ranges of the two sensors are in the form of D-S belief structures, as shown in Figure 8. For example, in Figure 8, the item m([3.7,3.9])=0.2, m([3.8,4.1])=0.8 indicates that the detection range is that of ambiguity composed of discord and non-specificity, where the distribution of beliefs 0.2 and 0.8 expresses the discord, and both the intervals [3.7, 3.9] and [3.8, 4.1] express the non-specificity. By facing the situation, the method of matrix games with payoffs of D-S belief structures will be used to compare the performance of the sensors in different task cases. Similarly, if the sensors are used to construct a linear barrier, each matrix in Figure 8 is treated as the payoff matrix in a game, and the value of the game becomes an equivalent detection range. By using the proposed solution method in Section 4, the value of the sensor games with D-S belief structure payoffs can be calculated, they are still D-S belief structures indicated by $m_{V_{A}}$ and $m_{V_{B}}$:

$$\begin{array}{c} m_{V_{A}}\left(\left[1.1648,1.2776\right]\right)=0.02,\\ m_{V_{A}}\left(\left[1.2109,1.3650\right]\right)=0.18,\\ m_{V_{A}}\left(\left[1.1745,1.2983\right]\right)=0.08,\\ m_{V_{A}}\left(\left[1.2214,1.3887\right]\right)=0.72; \end{array}$$

and

$$\begin{array}{c} m_{V_{B}}\left(\left[1.2500,1.5000\right]\right)=0.01,\\ m_{V_{B}}\left(\left[1.2737,1.5444\right]\right)=0.18,\\ m_{V_{B}}\left(\left[1.3000,1.6000\right]\right)=0.81. \end{array}$$

**Figure 9:**
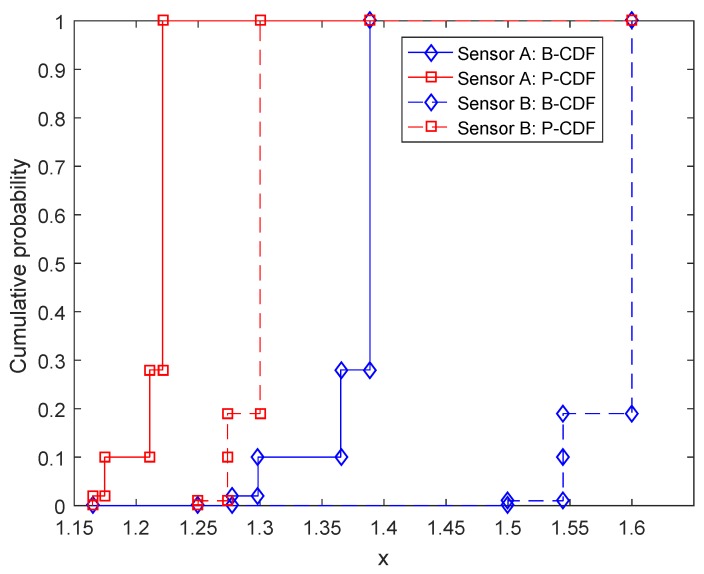
CDFs of the values of sensor games given in Figure 8 with respect to the associated variable *x*.

The associated CDFs of $m_{V_{A}}$ and $m_{V_{B}}$ are illustrated in Figure 9.

In order to compare the performance of Sensors A and B, $m_{V_{A}}$ and $m_{V_{B}}$ herein are regarded as distributions of interval numbers, then the expected detection ranges of Sensors A and B are $E\left(m_{V_{A}}\right)=\left[1.2146,1.3750\right]$ and $E\left(m_{V_{B}}\right)=\left[1.2948,1.5890\right]$. To compare $E(m_{V_{A}})$ and $E(m_{V_{B}})$, a simple heuristic method proposed by Wang et al. [77] is utilized in this paper, which provides the degree of possibility that an interval is greater/lesser than another one. In [77], for intervals $A=[a^{L},a^{U}]$ and $B=[b^{L},b^{U}]$, the possibilities of A≥B and B≥A are defined as below:

(16) $$P\left(A\geq B\right)=\frac{\max\left\{0,a^{U}-b^{L}\right\}-\max\left\{0,a^{L}-b^{U}\right\}}{a^{U}-a^{L}+b^{U}-b^{L}},$$

(17) $$P\left(B\geq A\right)=\frac{\max\left\{0,b^{U}-a^{L}\right\}-\max\left\{0,b^{L}-a^{U}\right\}}{a^{U}-a^{L}+b^{U}-b^{L}}.$$

According to Equations (16) and (17), we have $P\left(E\left(m_{V_{A}}\right)\geq E\left(m_{V_{B}}\right)\right)=0.1765$ and $P\left(E\left(m_{V_{B}}\right)\geq E\left(m_{V_{A}}\right)\right)=0.8235$. Therefore, Sensor B is more likely to perform better in the task of constructing linear barriers.

**Figure 10:**
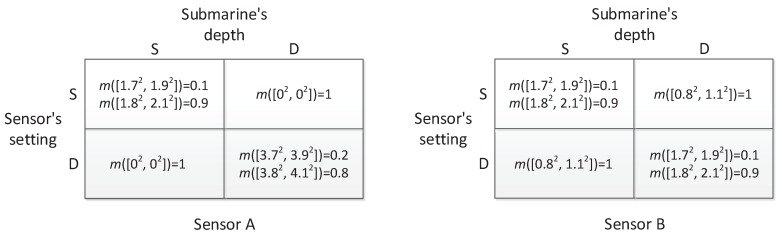
Squared detection ranges for Sensors A and B in the form of D-S belief structures.

In the other case, the sensors are used to detect hidden submarines within a large area. For Sensors A and B, since the task is changed to detecting submarines within an area to construct a linear barrier (in other words, from the barrier coverage to the area coverage), the games now are on the basis of squared detection ranges, as shown in Figure 10. Similarly, by using the proposed method in this paper, we have the values of the games in Figure 10, denoted as $m_{V_{A}^{\prime }}$ and $m_{V_{B}^{\prime }}$:

$$\begin{array}{c} m_{V_{A}^{\prime }}\left(\left[2.3863,2.9175\right]\right)=0.02,\\ m_{V_{A}^{\prime }}\left(\left[2.6199,3.4188\right]\right)=0.18,\\ m_{V_{A}^{\prime }}\left(\left[2.4081,2.9718\right]\right)=0.08,\\ m_{V_{A}^{\prime }}\left(\left[2.6462,3.4935\right]\right)=0.72; \end{array}$$

and

$$\begin{array}{c} m_{V_{B}^{\prime }}\left(\left[1.7650,2.4100\right]\right)=0.01,\\ m_{V_{B}^{\prime }}\left(\left[1.8462,2.5814\right]\right)=0.18,\\ m_{V_{B}^{\prime }}\left(\left[1.9400,2.8100\right]\right)=0.81. \end{array}$$

**Figure 11:**
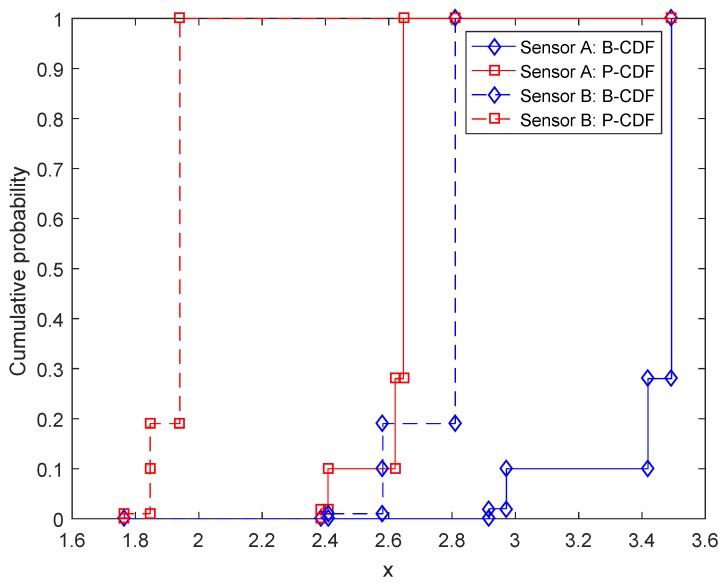
CDFs of the values of sensor games given in Figure 10 with respect to the associated variable *x*.

Likewise, the associated CDFs of $m_{V_{A}^{\prime }}$ and $m_{V_{B}^{\prime }}$ are shown in Figure 11. Then, we calculate the expected values of the games, which are $E\left(m_{V_{A}^{\prime }}\right)=\left[2.6172,3.4268\right]$ and $E\left(m_{V_{B}^{\prime }}\right)=\left[1.9214,2.7648\right]$. At last, according to Equations (16) and (17), it is derived that $P\left(E\left(m_{V_{A}^{\prime }}\right)\geq E\left(m_{V_{B}^{\prime }}\right)\right)=0.9107$ and $P\left(E\left(m_{V_{B}^{\prime }}\right)\geq E\left(m_{V_{A}^{\prime }}\right)\right)=0.0893$. Hence, it is well-founded to conclude that Sensor A is better than Sensor B for the task of detecting submarines within a large area.

Through the application, we can find that in the real world, sometimes, it indeed requires that the game model has the ability to handle uncertain payoffs. On the basis of D-S theory’s capability to represent the ambiguity involved in game payoffs, the proposed zero-sum matrix game with belief structure payoffs provides an effective way to meet this requirement.

### 6.2. Another Application on the Intrusion Detection in Sensor Networks

In this subsection, another example about the intrusion detection in sensor networks is given to illustrate the application and effectiveness of the proposed model.

The intrusion detection (ID) is of great concern in various kinds of sensor networks [78]. Currently, the ID techniques are mainly classified into misuse detection and anomaly detection. Generally, the misuse detection is usually used to detect known types of attacks whose attack characteristics (known as “signatures”) are known and could achieve high detection accuracy and a low false alarm rate for such attacks, but is unable to detect novel attacks. In contrast, the anomaly detection has good performance in detecting novel attacks, but it is inclined to a high false alarm rate. The signature-based intrusion detection system (IDS) and anomaly-based IDS are two typical IDSs for misuse detection and anomaly detection, respectively. Therefore, integrating multiple detection techniques is a promising way to improve overall detection performance. In [79], the authors have presented a game-theoretic framework to study the interactions between attackers and defenders in the ID by integrating various ID techniques. They assumed that a defender can use multiple IDSs and an attacker can stage multiple types of attacks. In the paper, the two-person zero-sum game presented in [79] is utilized to show the practicable application of the proposed zero-sum matrix game with belief structure payoffs.

In [79], it is assumed that a sensor network could deploy multiple weak IDSs in which “weak” means that each IDS has different limitations in its coverage of attack types and has different performances in detecting different types of attacks. Particularly, suppose an attacker is able to stage two attacks on a sensor network, Attack 1 and Attack 2, so it has three strategies: Attack 1, Attack 2 and not attack. In response, a defender can use two IDSs, for example signature-based IDS and anomaly-based IDS, against the attacker: IDS1, which performs better for Attack 1, and IDS2, having better performance for Attack 2. Hence the defender also has three strategies: IDS1, IDS2 and not monitor. As a result, the defender and attacker constitute a 3×3 two-person zero-sum game; the payoff matrix of the defender is shown in Table 3. The payoffs are explained as follows. Let $\alpha_{ij}$ and $1-\alpha_{ij}$ be the detection rate and false negative rate of the *i*-th IDS with respect to the *j*-th attack type and $\beta_{i}$ the false positive rate of the *i*-th IDS, where $\alpha_{ij},\beta_{i}\in \left[0,1\right]$, and *h* represents the merit of the overall network. The payoff function of the defender, denoted as $U_{ij}$, is defined by:

$$U_{ij}=\left\{\begin{array}{c} (2\alpha_{ij}-1)h,\;i,j=1,2\\ -h,\;\;\;\;i=3,j=1,2\\ -\beta_{i}h,\;\;\;\;i=1,2,j=3\\ 0,\;\;\;\;\;\;\;i=3,j=3 \end{array}\right.$$

where $(2\alpha_{ij}-1)h$ is the expected gain of detecting the *j*-th attack type by the *i*-th IDS, which is calculated by the detection gain of the defender $\alpha_{ij}h$ plus the loss of a failed detection $-(1-\alpha_{ij})h$. Additionally, $-\beta_{i}h$ represents the loss (cost) of false alarm attacks when only normal data are presented. Since the game is zero-sum, the payoff of the attacker is $-U_{ij}$. In the constructed attacker/defender game, the equilibrium strategy shows the contribution of each IDS to the overall sensor network security, which can be used as a reference to further improve the IDS configuration. Additionally, the value of the game, denoted as *V*, represents a minimum security level (MSL) the defender can achieve; it is a key indicator of the sensor network security.

A numerical example is given in [79] by setting specific values for the parameters mentioned above. Let $\alpha_{11}=85\%$, $\alpha_{12}=45\%$, $\beta_{1}=0.1\%$, $\alpha_{21}=20\%$, $\alpha_{22}=70\%$, $\beta_{2}=2\%$ and h=100, then a numerical payoff matrix of the attacker/defender game is generated:

$$U_{defender}=\left[\begin{array}{ccc} 70 & -10 & -0.1\\ -60 & 40 & -2\\ -100 & -100 & 0 \end{array}\right].$$

For the above $U_{defender}$, the value of the game is V=−0.4624, which means that the MSL of the corresponding sensor network is −0.4624.

**Figure 12:**
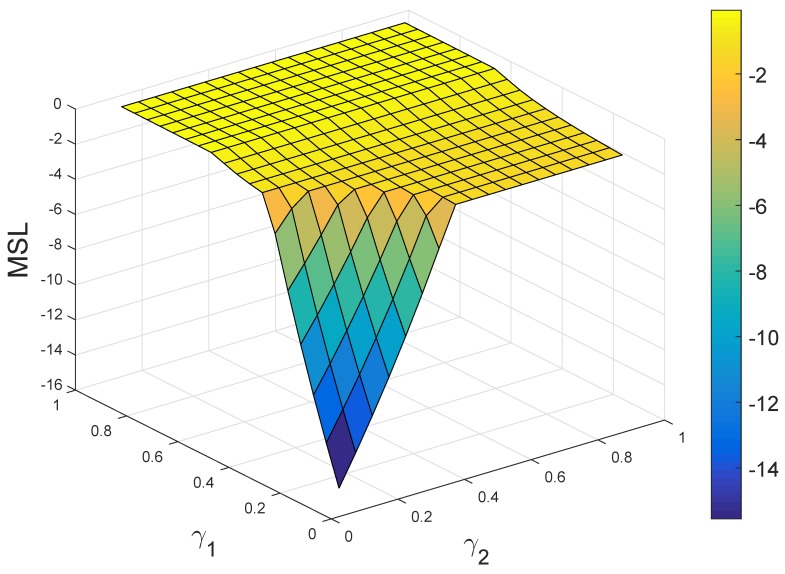
The minimum security level (MSL) of the sensor network with respect to parameters $\gamma_{1}$ and $\gamma_{2}$ in terms of $U_{defender}^{\prime }$.

Clearly, in the above numerical example, it is assumed that IDS1 has a bad performance with respect to Attack 2 (namely $\alpha_{12}=45\%$) and IDS2 performs very poorly to Attack 1 (namely $\alpha_{21}=20\%$). In order to elevate the overall security level of the sensor network, a straightforward way is to improve the performances of IDS1 to Attack 2 and IDS2 to Attack 1, respectively. Now, we analyze the effect of such improvement. In order to decrease the number of parameters, we assume $\alpha_{12}=\gamma_{1}\alpha_{11}$ and $\alpha_{21}=\gamma_{2}\alpha_{22}$, where $\gamma_{1},\gamma_{2}\in \left[0,1\right]$. Therefore, the payoff matrix of the defender now becomes:

(18) $$U_{defender}^{\prime }=\left[\begin{array}{ccc} (2\alpha_{11}-1)h & (2\gamma_{1}\alpha_{11}-1)h & -\beta_{1}h\\ (2\gamma_{2}\alpha_{22}-1)h & (2\alpha_{22}-1)h & -\beta_{2}h\\ -h & -h & 0 \end{array}\right]$$

where $\alpha_{11}=85\%$, $\alpha_{22}=70\%$, $\beta_{1}=0.1\%$, $\beta_{2}=2\%$ and h=100. According to $U_{defender}^{\prime }$, the MSL of the sensor network can be obtained with respect to different $\gamma_{1}$ and $\gamma_{2}$, as shown in Figure 12. From Figure 12, it is found that: (i) if both the values of $\gamma_{1}$ and $\gamma_{2}$ are small, increasing them can obviously elevate the MSL of the sensor network; (ii) however, if $\gamma_{1}$ or $\gamma_{2}$ has already obtained a big value, increasing the value of the other parameter cannot observably improve the security of the sensor network. The results are very useful in the process of improving IDS1 and IDS2 to avoid unnecessary work and save cost. What is more, from Figure 12, it seems that the MSL almost reaches the highest level only if $\gamma_{1}\geq 0.6$, regardless of the value of $\gamma_{2}$. This is obtained from the analysis of the attacker/defender game with deterministic payoffs.

However, in the above analysis, the uncertainty is not taken into consideration in the process of improving IDS1 and IDS2. For example, if $\gamma_{1}=0.55$ resulting in $\alpha_{12}=46.75\%$, it actually assumes that the improved IDS1 has an exact 46.75% detection rate with respect to Attack 2. In reality, a security architect may hardly ensure that an improved IDS has an exact detection rate. Instead, the performance index of the improved IDS is usually of uncertainty, for instance lying in an interval $\alpha_{12}\in \left[45\%,47\%\right]$. Dose such uncertainty have a severe impact on the security of the sensor network? This problem can be addressed by using the proposed model in the paper. A numerical example is given first as follows.

If $\gamma_{1}=0.55$ and $\gamma_{2}=0.7$, it is easily obtained that the MSL of the sensor network is V=−0.3512 in terms of Equation (18). Now, suppose that $\gamma_{1}$ and $\gamma_{2}$ are uncertain, which are represented by belief structures:

$$\begin{array}{c} m_{\gamma_{1}}\left(\left[0.53,0.56\right]\right)=0.5,\;m_{\gamma_{1}}\left(\left[0.54,0.57\right]\right)=0.5;\\ m_{\gamma_{2}}\left(\left[0.68,0.71\right]\right)=0.5,\;m_{\gamma_{2}}\left(\left[0.69,0.72\right]\right)=0.5. \end{array}$$

Herein, the mean values of $\gamma_{1}$ and $\gamma_{2}$, expressed by $m_{\gamma_{1}}$ and $m_{\gamma_{2}}$, are approximately equal to 0.55 and 0.7, respectively. According to Equation (18), a new zero-sum matrix games with belief structure payoffs can be generated, in which the payoff matrix of the defender is shown in Table 4. Then, in terms of the proposed solution method, the value of the game in Table 4 can be obtained:

$$\begin{array}{c} m_{V}\left(\left[-0.4595,-0.2912\right]\right)=0.25,\\ m_{V}\left(\left[-0.4072,-0.2267\right]\right)=0.25,\\ m_{V}\left(\left[-0.4595,-0.2912\right]\right)=0.25,\\ m_{V}\left(\left[-0.4072,-0.2267\right]\right)=0.25. \end{array}$$

Additionally, the expected value of $m_{V}$ is $E\left(m_{V}\right)=\left[-0.4333,-0.2589\right]$. Therefore, the MSL of the sensor network becomes an interval [−0.4333,−0.2589] from a crisp number −0.3512, because of the consideration of uncertainty. Here, we define a fluctuation coefficient *Q* to express the impact of considering such uncertainty:

$$Q=\left|\frac{range(E\left(m_{V}\right))}{V}\right|\times 100\%.$$

In the numerical example, the value of *Q* is calculated as 49.65%, which indicates that the MSL has relatively large fluctuations in the case of considering the uncertainty involved in $\gamma_{1}$ and $\gamma_{2}$, compared with its deterministic case. Therefore, according to the result obtained by using our proposed model, in this numerical example, the uncertainty has to be cautiously considered in the process of improving IDS1 and IDS2. The security architect is suggested to try the best to make $\gamma_{1}$ and $\gamma_{2}$ more precise in order to achieve the expected MSL shown in Figure 12.

**Figure 13:**
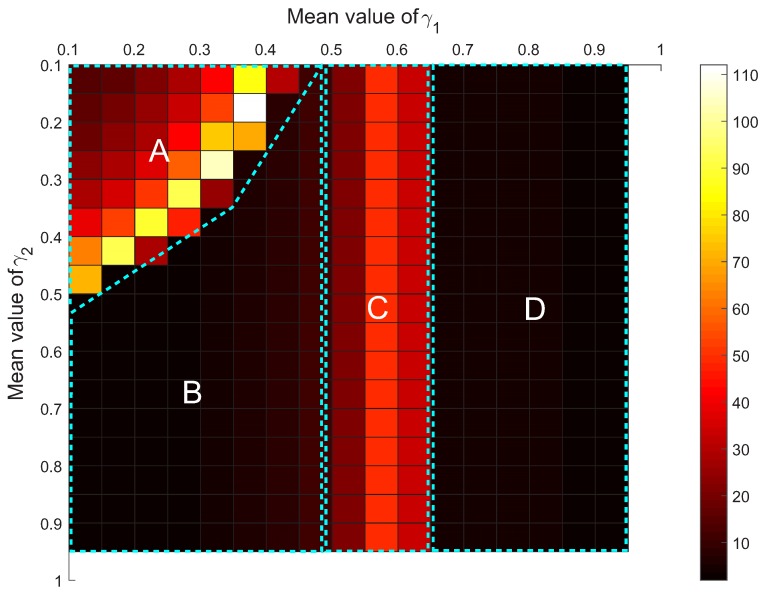
The value of fluctuation coefficient *Q* with respect to different mean values of $\gamma_{1}$ and $\gamma_{2}$.

By this means, all *Q* values associated with different mean values of $\gamma_{1}$ and $\gamma_{2}$ can be derived, as shown in Figure 13. In the process, for each deterministic value of $\gamma_{1}$ or $\gamma_{2}$, denoted as γ, it is transformed to a Dempster–Shafer belief structure via the following means:

m([γ−l,γ+l/2])=0.5,m([γ−l/2,γ+l])=0.5

where *l* represents the radius of the uncertainty of γ. From Figure 13, the distribution map of the value of *Q* can be classified into four areas, A, B, C and D. In Areas B and D, each value of *Q* is very small, which means that the uncertainty in $\gamma_{1}$ and $\gamma_{2}$ basically has no influence on the MSL of the sensor network compared with the case that $\gamma_{1}$ and $\gamma_{2}$ get deterministic values. In contrast, in Areas A and C the uncertainty expressed by $m_{\gamma_{1}}$ and $m_{\gamma_{2}}$ distinctly causes the MSL to fluctuate to different extents. In Area A, the uncertainty both in $\gamma_{1}$ and $\gamma_{2}$ can impact the final MSL, and with the increase of $\gamma_{1}$ or $\gamma_{2}$, the fluctuation becomes more and more severe. Therefore, the security architect should prevent IDS1 and IDS2 from falling into this area as much as possible. In Area C, it is found that only $\gamma_{1}$’s uncertainty influences the final MSL; therefore, the security architect should give the most attention to the process of improving IDS1 to reduce the uncertainty of $\alpha_{12}$. By synthetically considering Figure 12 and Figure 13, if letting $\gamma_{1}\geq 0.65$ and $\gamma_{2}\geq 0.45$ (i.e., improving IDS1 and IDS2 to implement $\alpha_{12}\geq 55.25\%$ and $\alpha_{21}\geq 31.5\%$), on the one hand, the sensor network can achieve the highest security level that IDS1 and IDS2 can provide; on the other hand, the security level can basically remain unchanged with the possible uncertainty of IDS1’s detection rate for Attack 2 and IDS2’s detection rate for Attack 1. The result gives the security architect a more practical and effective suggestion of how to optimize the IDS configuration of the sensor network. Through the example of the intrusion detection of sensor networks, it shows another potential application of the proposed model, which is helpful for improving the security of sensor networks.

## 7. Conclusions

In this paper, zero-sum matrix games with uncertain payoffs have been addressed. In order to express the ambiguity involved in the payoffs of a game, D-S theory has been used to represent the uncertain payoffs. As a result, a zero-sum matrix game with payoffs of D-S belief structures has been presented. In order to obtain the value of such a game, in the paper, a decomposition method is proposed by transforming the game into multiple zero-sum matrix games with interval data. Moreover, for the possible computationally-intensive issue in the process of applying the decomposition method, as an alternative solution, a Monte Carlo simulation approach based on LHS is provided, as well. Finally, applications of the proposed game model and its solution method in sensor selection and intrusion detection of sensor networks are given, which show the usefulness and effectiveness of the novel game model. In future research, the authors will invest more effort into seeking other potential applications so as to further improve the proposed game model.

## Figures and Tables

**Table 1:** A 2×2 zero-sum matrix game with D-S belief structure payoffs.

Strategy	$\sigma_{1}$	$\sigma_{2}$
$\delta_{1}$	$\begin{array}{c} m([1,2])=0.6\\ m([3,5])=0.4 \end{array}$	$\begin{array}{c} m([-1,3])=1 \end{array}$
$\delta_{2}$	$\begin{array}{c} m([-1,0])=1 \end{array}$	$\begin{array}{c} m([-1,1])=0.2\\ m([0,2])=0.8 \end{array}$

**Table 2:** A 5×3 zero-sum matrix game with D-S belief structure payoffs.

	$\sigma_{1}$	$\sigma_{2}$	$\sigma_{3}$
$\delta_{1}$	$\begin{array}{c} m([1,2])=0.5,m([0,2])=0.1,\\ m([3,5])=0.3,m([4,5])=0.1 \end{array}$	$\begin{array}{c} m([-1,1])=0.5,m([-1,2])=0.3,\\ m([-1,3])=0.1,m([2,3])=0.1 \end{array}$	$\begin{array}{c} m([-3,2])=0.4,m([-1,1])=0.3,\\ m([-2,2])=0.1,m([1,2])=0.2 \end{array}$
$\delta_{2}$	$\begin{array}{c} m([-1,0])=0.2,m([-1,1])=0.3,\\ m([0,3])=0.3,m([1,2])=0.2 \end{array}$	$\begin{array}{c} m([-1,1])=0.2,m([0,1])=0.2,\\ m([0,1.5])=0.3,m([1,2])=0.3 \end{array}$	$\begin{array}{c} m([-2,-1.5])=0.1,m([-1,1])=0.3,\\ m([0,2])=0.3,m([1,3])=0.3 \end{array}$
$\delta_{3}$	$\begin{array}{c} m([-1,0])=0.2,m([-1,1])=0.3,\\ m([1,2])=0.1,m([0,2])=0.4 \end{array}$	$\begin{array}{c} m([-2,0])=0.2,m([-1,0])=0.2,\\ m([-0.5,1])=0.3,m([0,1])=0.3 \end{array}$	$\begin{array}{c} m([-1,0])=0.3,m([-0.5,0])=0.2,\\ m([1,2])=0.3,m([0,2])=0.2 \end{array}$
$\delta_{4}$	$\begin{array}{c} m([-1,2])=0.1,m([0,2])=0.7,\\ m([0,1])=0.1,m([1,2])=0.1 \end{array}$	$\begin{array}{c} m([-2,0])=0.4,m([-1,1])=0.1,\\ m([-0.5,1])=0.2,m([0,1])=0.3 \end{array}$	$\begin{array}{c} m([-1.5,1])=0.3,m([-0.5,0])=0.1,\\ m([-1,2])=0.1,m([1,3])=0.5 \end{array}$
$\delta_{5}$	$\begin{array}{c} m([-2,-1])=0.4,m([-1,1])=0.2,\\ m([0,1])=0.3,m([1,2])=0.1 \end{array}$	$\begin{array}{c} m([-3,1])=0.6,m([-1,2])=0.1,\\ m([1,2])=0.2,m([0,2])=0.1 \end{array}$	$\begin{array}{c} m([-2,-1])=0.4,m([-1,1])=0.2,\\ m([-1,2])=0.2,m([1,3])=0.2 \end{array}$

**Table 3:** The payoff matrix of the defender in a zero-sum attacker/defender game.

	Attack 1	Attack 2	Not Attack
IDS1	$(2\alpha_{11}-1)h$	$(2\alpha_{12}-1)h$	$-\beta_{1}h$
IDS2	$(2\alpha_{21}-1)h$	$(2\alpha_{22}-1)h$	$-\beta_{2}h$
Not monitor	−h	−h	0

**Table 4:** The payoff matrix of the defender in a zero-sum attacker/defender game with belief structure payoffs. IDS, intrusion detection system.

	Attack 1	Attack 2	Not Attack
IDS1	m([70,70])=1	$\begin{array}{c} m([-9.9,-4.8])=0.5\\ m([-8.2,-3.1])=0.5 \end{array}$	m([−0.1,−0.1])=1
IDS2	$\begin{array}{c} m([-4.8,-0.6])=0.5\\ m([-3.4,0.8])=0.5 \end{array}$	m([40,40])=1	m([−2,−2])=1
Not monitor	m([−100,−100])=1	m([−100,−100])=1	m([0,0])=1
